# Somatosensory cortex neuronal integrity is altered after stroke

**DOI:** 10.3389/fnhum.2026.1810922

**Published:** 2026-05-20

**Authors:** Rafay Cheema, Nawal Cheema, Andrew Apostol, Mojgan Golzy, Mihaela Carmen Cirstea

**Affiliations:** 1 Boston University, Boston, MA, United States; 2 School of Medicine, Department of Physical Medicine and Rehabilitation, University of Missouri, Columbia, MO, United States; 3 Department of Biomedical Informatics, Biostatistics, and Medical Epidemiology, School of Medicine, University of Missouri, Columbia, MO, United States; 4 Department of Neurology, School of Medicine, Kansas University Medical Center, Kansas City, KS, United States

**Keywords:** subcortical stroke, somatosensory cortex, N-acetylaspartate, glutamate-glutamine complex, hand impairment

## Abstract

**Background:**

Sensorimotor remapping plays a crucial role in hand function rehabilitation after stroke. While motor remapping has been intensively investigated at various levels, from functional to metabolic, limited attention has been given to somatosensory cortical remapping. This study extends our previous work in stroke, showing functionally relevant metabolic alterations in radiologically normal-appearing or spared motor cortices, predominantly in the ipsilesional (stroke-injured) hemisphere. We investigated the metabolic status of the ipsilesional primary somatosensory cortex (S1) and its relation to hand impairment after a stroke.

**Methods:**

Fourteen individuals with a subacute-to-chronic ischemic subcortical stroke leading to a moderate hand/arm motor impairment (Fugl-Meyer Upper Extremity, Jamar dynamometer), but without sensory deficits (Semmes-Weinstein monofilaments), and 10 matched healthy controls participated. MR Spectroscopy markers of neuronal integrity (N-acetylaspartate) and neuronal-glial glutamatergic cycle/neurotransmission (glutamate-glutamine complex) were measured in the ipsilesional radiologically normal-appearing S1 hand territory (3 T, Allegra Siemens Medical Solutions, Erlangen, Germany). Multivariate models (SAS v 9.4) with covariates such as age, sex, and time since stroke were used for the entire patient group. A patient subgroup analysis (*n* = 7 in each) was also performed: subacute (≤6 months after onset) and chronic (>6 months).

**Results:**

Compared with controls, the patient group showed significantly lower N-acetylaspartate levels, with greater alterations in the subacute subgroup. No significant alterations in glutamate-glutamine levels were found in patients. N-acetylaspartate was significantly correlated with glutamate-glutamine in patients, but not in controls. Significant correlations between N-acetylaspartate and hand impairment were found in those with chronic stroke.

**Conclusion:**

Our preliminary findings suggest neuronal mitochondrial dysfunction, which correlates with reduced cortical excitability in the ipsilesional S1 across subacute and chronic phases of stroke. S1 neuronal dysfunction positively correlates with hand impairment in the chronic but not in the subacute phase of disease. Future longitudinal work with larger sample sizes is warranted.

## Introduction

Recovery of hand motor function after a stroke involves a dynamic process that engages brain areas beyond the motor system (Nudo, 2011). One of these areas is the primary somatosensory cortex (S1), which is well known for its involvement in sensorimotor integration and feedback mechanisms, supporting the planning, execution, and learning of motor skills (Bernardi et al., 2013; Bernardi et al., 2015; Gale et al., 2021; Mountcastle and Sensory, 2005; Sasaki et al., 2017). Nevertheless, the last two decades of stroke rehabilitation research have focused on motor remapping or reorganization, particularly of the primary motor cortex (M1, a critical cortical area responsible for hand motor control; Dum and Strick, 1996; Omrani et al., 2017) and recovery (Nudo, 2011; Nudo et al., 1996), leaving S1 remapping understudied (see review Edwards et al., 2019). Few studies demonstrated the crucial role of sensorimotor interactions in hand rehabilitation after a unilateral stroke. For example, in the early weeks after stroke, there is a shift in S1 activation (as measured by functional MRI) toward the contralesional, or non-injured, hemisphere (Carson, 2020; Williamson et al., 2024). This early functional reorganization is followed by a switch back to, or “normalization” of, S1 activation in the ipsilesional, or injured, hemisphere, a process that parallels recovery of skilled hand movements (Williamson et al., 2024). Non-invasive S1 stimulation also promotes recovery of hand movements in all phases of stroke (Brodie et al., 2014; de Freitas Zanona et al., 2022). Briefly, repetitive transcranial magnetic stimulation over the S1, paired with motor practice or sensory stimulation, is likely to enhance learning and recovery of hand movements. Likewise, sensory stimulation alone appears to increase corticospinal excitability and enlarge hand motor representation, ultimately promoting hand recovery (see review Serrada et al., 2019). Despite growing evidence of S1’s role in the rehabilitation of hand movements, no study has investigated whether its metabolic status is altered and how these alterations may contribute to hand impairment or recovery after a stroke. Such understanding may lead to the identification of new targets for hand impairment-focused restorative therapies (Lai et al., 2002).

Our group was the first to characterize the nature, evolution, and functional relevance of MR Spectroscopy (MRS)-detected metabolic alterations across motor cortices during different phases of stroke (Anunoby et al., 2009; Austin et al., 2021; Cirstea et al., 2011; Cirstea et al., 2018; Cirstea et al., 2014; Cirstea et al., 2012; Craciunas et al., 2013). Specifically, in the radiologically normal-appearing M1 controlling the paretic hand (ipsilesional), we found lower levels of the putative marker of neuronal integrity, N-acetylaspartate (NAA), supporting the likelihood of widespread neuronal metabolic downregulation in remote areas connected to the injury site, so-called diaschisis (Jia et al., 2026; Pascoa Dos Santos and Verschure, 2021; Seitz et al., 1999). Changes in MRS-detected markers of the balance between excitatory neurotransmission and glial recycling pathways (Andersen, 2025), i.e., the glutamate-glutamine complex (or Glx), further support this phenomenon: lower Glx levels suggesting a dysregulation of excitatory signaling in ipsilesional M1. Notably, the NAA and Glx alterations are functionally relevant, (i) relating to stroke chronicity and severity (Cirstea et al., 2011; Craciunas et al., 2013), (ii) predicting hand motor outcomes with interventions (Cirstea et al., 2018), and (iii) normalizing when hand movements recover (Austin et al., 2021). We also found altered correlational patterns between these two markers; a higher, positive, and significant correlation was observed between NAA and Glx compared to those in the healthy population (Craciunas et al., 2013), indicating a strong relationship between neuronal metabolic status and excitability of that area. This, together with our finding of a significant correlation between NAA levels and the spatial extent of M1 activation during a task performed with the paretic hand (Cirstea et al., 2014), sheds light on the potential neural mechanisms underlying the well-known ipsilesional M1 hypoexcitability after a stroke (Stinear et al., 2015). Whether similar alterations in the levels and/or correlations of these markers occur in ipsilesional spared S1 and how these alterations relate to hand impairment are unknown.

We hypothesized that participants with subcortical stroke would exhibit altered levels of NAA and Glx in the ipsilesional S1 hand territory, as these alterations have been observed in the ipsilesional M1 in a similar population (Cirstea et al., 2011), and given the extensive anatomical connections between S1 and M1 (Gandolla et al., 2014; Papale and Hooks, 2018; Rocco-Donovan et al., 2011). However, given differences in cellularity and function (see the review Edwards et al., 2019), a different magnitude of these alterations could be expected compared to those in M1. Because the cellular mechanisms relevant to brain repair in the subacute phase of stroke (defined here as less than 6 months after onset) are not relevant at later time points (Biernaskie et al., 2004), we would also expect metabolite alterations of different magnitudes across stroke phases. We further hypothesized that metabolite correlations would be altered similarly to those observed in M1 (Cirstea et al., 2011), with NAA and Glx showing greater correlation than in controls. Finally, we expected to find a significant relationship between these metabolite alterations and the clinically assessed hand motor impairment, reflecting a possible role in post-stroke motor impairment.

## Materials and methods

### Participants

Fourteen chronic stroke survivors and 10 age- and sex-matched healthy controls provided informed consent in accordance with the University of Kansas Medical Center Institutional Review Board. Stroke participants were included if they: (i) had experienced a single ischemic stroke at the subcortical level resulting in unilateral hemiparesis at least 1 month before participation, (ii) had no lesion in S1 or thalamus on T2-weighted MRI, (iii) had normal hand sensation (as detected by Semmes-Weinstein monofilaments; Suda et al., 2020), and (iv) sufficient motor function to complete a handgrip task (Fugl-Meyer Upper Extremity Scale; Duncan et al., 1983, FMUE, greater than 10) since these patients participated to a motor intervention. The data reported here were collected at the baseline (before the intervention). Participants with contraindications to MRI or comorbid neurological, psychiatric, or orthopedic conditions were excluded from the study. Ten healthy controls, free of neurologic, psychiatric, or systemic disorders and demonstrating normal structural MRI findings, were included in the analysis.

### Experimental protocol

#### Hand impairment evaluation

The Fugl-Meyer Upper Extremity (FMUE, normal = 66) scale was used to quantify arm and hand motor impairment. Hand strength was assessed bilaterally using a Jamar dynamometer (Asimow Engineering Co., Los Angeles, CA), and the strength of the paretic hand was expressed as a percentage of the non-paretic hand strength (normal > 89%).

#### MRS imaging and analysis

**Figure 1:**
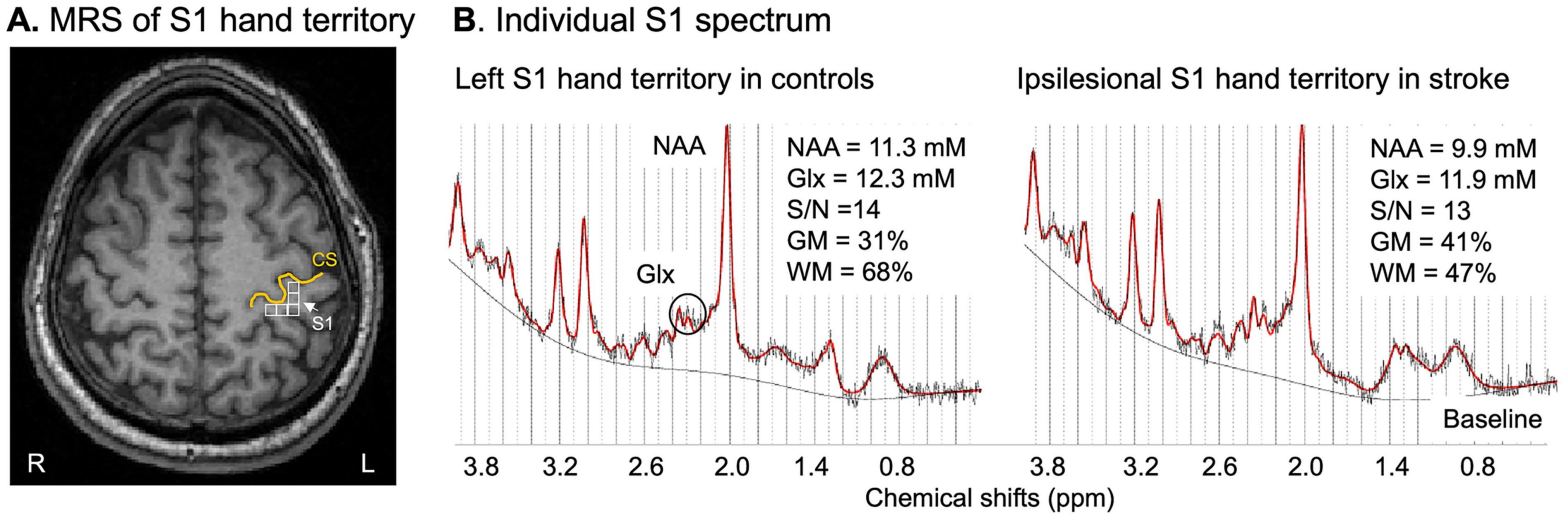
**(A)** MRS voxels (white squares) located in the S1 hand territory on high-resolution T1 MPRAGE in a control participant; R, right, L, left, orange line follows the central sulcus (CS). **(B)** LCModel output from a spectroscopic voxel in the left (in a control participant) and ipsilesional (in a patient) S1 hand territories. The output included the acquired MRS spectrum (black line), the estimated spectrum (red line), and the underlying baseline. Spectra qualities were high, with a signal-to-noise (S/N) of 14 for the control and 13 for the patient. NAA and Glx concentrations were calculated (LCModel) and corrected for the brain tissue composition (% grey matter, GM + % white matter, WM) within the spectroscopic voxel. ppm, parts per million.

Neuroimaging data were collected using a 3 T Siemens MRI scanner. During scanning, the heads of all participants were immobilized with head cushions and instructed not to move. A high-resolution T1-weighted anatomical scan (MPRAGE; TR = 2,300 ms; TE = 3 ms; matrix = 256 × 256; voxel size = 1 × 1 × 1 mm^3^) was acquired to guide MRS slab placement and estimate brain tissue composition within the spectroscopic voxels. Axial T2-weighted and proton density images (TR = 4,800 ms; TE1/TE2 = 18/106 ms; slice thickness = 5 mm, no gap) were used to verify that the S1 remained radiologically intact and confirm the subcortical stroke location. MRS imaging was performed using a point-resolved spectroscopy (PRESS) sequence (TE = 30 ms; TR = 1,500 ms; voxel size = 15 × 15 × 15 mm^3^; spectral width = 1,200 Hz). Based on anatomical landmarks, a multivoxel slab was placed across the ipsilesional sensorimotor cortices: the hand S1 territory voxels were specifically located on the posterior bank of the central sulcus across the primary motor cortex hand knob (Figure 1A).

Eight 20 mm outer volume suppression bands were used to minimize lipid contamination. Automated, followed by manual, shimming was performed to achieve an optimal full width at half maximum of <20 Hz for the water signal from the entire excitation volume. Motion correction was performed using a rigid-body transformation, estimating six parameters: three translational and three rotational. These parameters were inspected for head movement. None of the participants moved their head more than 2 mm in any direction. The total scan duration was about 45 min.

MRS analysis methods have been detailed previously (Cirstea et al., 2011) and consisted of MRS data [LCModel, a linear combination of model spectra using a basis set included in the package, and a radio-frequency coil loading factor (Provencher, 2001)] and T1-weighted image analysis (brain segmentation, SPM12, Welcome Department of Cognitive Neurology, London, United Kingdom). At least three spectroscopic voxels with the following characteristics, gray matter > 75%, a signal-to-noise ratio > 10, and Cramer-Rao lower bounds < 20%, were selected in the hand territory of ipsilesional S1. Using a custom-designed software (The Mathworks. Inc, 2023; Natick, Massachusetts), each voxel’s metabolite values were corrected for partial volume effects (Jansen et al., 2006) using the formula: c = cLCModel × [1/Fbrain], where Fbrain = % grey matter + % white matter within the spectroscopic voxel, derived from brain tissue segmentation in SPM12 (Wellcome Department of Cognitive Neurology, London, United Kingdom). The levels of each metabolite in the selected voxels were averaged and expressed in millimoles per kilogram of wet weight brain tissue. Since more than half of the patients had left hemisphere injury (57%) (Table 1), for those with the stroke located in the right hemisphere (*n* = 6), we right-to-left flipped the data so that the “left” hemisphere was the ipsilesional one. This flip was performed because there were no significant differences between hemispheres in MRS-detected metabolites in the healthy population (Cirstea et al., 2011; Cirstea et al., 2012). Thus, the ipsilesional S1 metabolites were compared with those in the left S1 in controls.

**Table 1:** Demographic and clinical data for stroke participants (*n* = 14), including age, sex (M, male; F, female), time since stroke (months, mo), affected hemisphere (L, left; R, right), stroke location, Fugl-Meyer Upper Extremity (FMUE, normal = 66) scores, and handgrip strength (expressed as a percentage of the non-paretic hand, normal > 89%) at recruitment. The patients are ordered by FMUE score, from least to most severe impairment.

Age/Sex	Time post-onset (mo)	Site of stroke	FMUE	Hand strength (%)
57/M	6	L/basal ganglia, corona radiata	66	91.5
48/F	1	R/corona radiata	66	91.2
71/F	5	R/corona radiata	66	89.3
68/M	2	L/posterior insula	66	91.6
75/M	1	L/internal capsule	62	98.3
65/M	3	R/basal ganglia	62	90.3
48/F	11	L/internal capsule, basal ganglia	61	93.6
45/F	27	R/basal ganglia, corona radiata	36	41.9
61/M	15	L/Basal ganglia	30	30.8
64/M	30	R/basal ganglia, internal capsule, corona radiata	27	3.7
66/F	2	L/internal capsule	26	8.8
59/M	144	R/internal capsule, basal ganglia	25	10.3
58/M	27	L/internal capsule, basal ganglia	24	8.6
61/M	24	L/basal ganglia, internal capsule, corona radiata	10	7.2

Highlighted participants form the subacute patient subgroup, while the remaining ones form the chronic subgroup.

#### Statistical analysis

Statistical analyses were performed using a combination of parametric and non-parametric tests, depending on data distribution (as determined by the Kolmogorov–Smirnov test). If data were non-normally distributed, a Mann–Whitney U test and Spearman correlation test were used. If normally distributed, a *t*-test and a Pearson correlation test were used. Group comparisons and correlation analyses were conducted between stroke participants and age, sex, and school-year-matched healthy controls. Multivariate models with covariates such as age and sex have also been used for metabolite-level comparison analyses, and time since stroke was included as a covariate to examine correlations between metabolites and clinical scores. Considering the broad post-stroke time frame, a similar analysis was conducted on two patient chronicity-based subgroups: subacute (≤6 months after onset, *n* = 7) and chronic (>6 months, *n* = 7). All analyses used SAS (v 9.4), and results were two-tailed with *α* = 0.05.

## Results

### Participants’ characteristics

Stroke and control participants did not significantly differ in age (mean ± SD = 60.4 ± 8.8 vs. 54.0 ± 10.8 yrs., *p =* 0.1), sex distribution (35.7% vs. 40.0% women, *p =* 0.4), or years of education (14.3 ± 2.0 vs. 14.9 ± 2.5; *p* = 0.6). All patients had sustained a single subcortical or brainstem infarction (in one patient) for at least 1 month before testing (Table 1) and had radiologically intact S1 on T2-weighted imaging. Patients demonstrated a wide range of arm/hand impairment (FMUE = 44.8 ± 20.9, from 10 to 66) and hand strength (54.1% ± 40.9% of the non-paretic side, from 3.7 to 98.3%) (Table 1). Subacute (2.7 ± 1.9 months after stroke onset, highlighted participants in Table 1) and chronic (41.4 ± 45.7 months) subgroups did not differ in terms of age (64.3 ± 9.1 vs. 56.6 ± 7.2 yrs., *p* = 0.10), sex distribution (3/7 vs. 2/7 women), or scholar years (14.9 ± 2.3 vs. 13.4 ± 1.9 yrs., *p* = 0.22). However, the subacute subgroup was less impaired than the chronic one (FMUE, 59.1 ± 14.7 vs. 33.3 ± 17.3, *p* = 0.01; hand strength: 80.1 ± 31.6% vs. 36.0 ± 39.1%, *p* = 0.04).

### Spectra quality were high in stroke and control groups

Signal-to-noise ratios for all spectra in each group were within recommended tolerances (11.6 ± 1.4 in patients, from LCModel vs. 12.7 ± 1.6 in controls, *p* = 0.62) (Figure 1B and Supplementary Table S1 for individual data). The full width at half maximum of the metabolites analyzed was about 12 Hz across all participants, indicating high spectral resolution.

### The brain tissue composition in the spectroscopic voxels was similar between stroke and control groups

Similar S1 brain tissue (grey matter plus white matter) compositions were found between groups (90.1 ± 4.4% in patients vs. 86.3 ± 6.5%, *p* = 0.13) (Supplementary Table S1 for individual data).

### Stroke participants exhibit significantly lower NAA levels than controls

**Figure 2:**
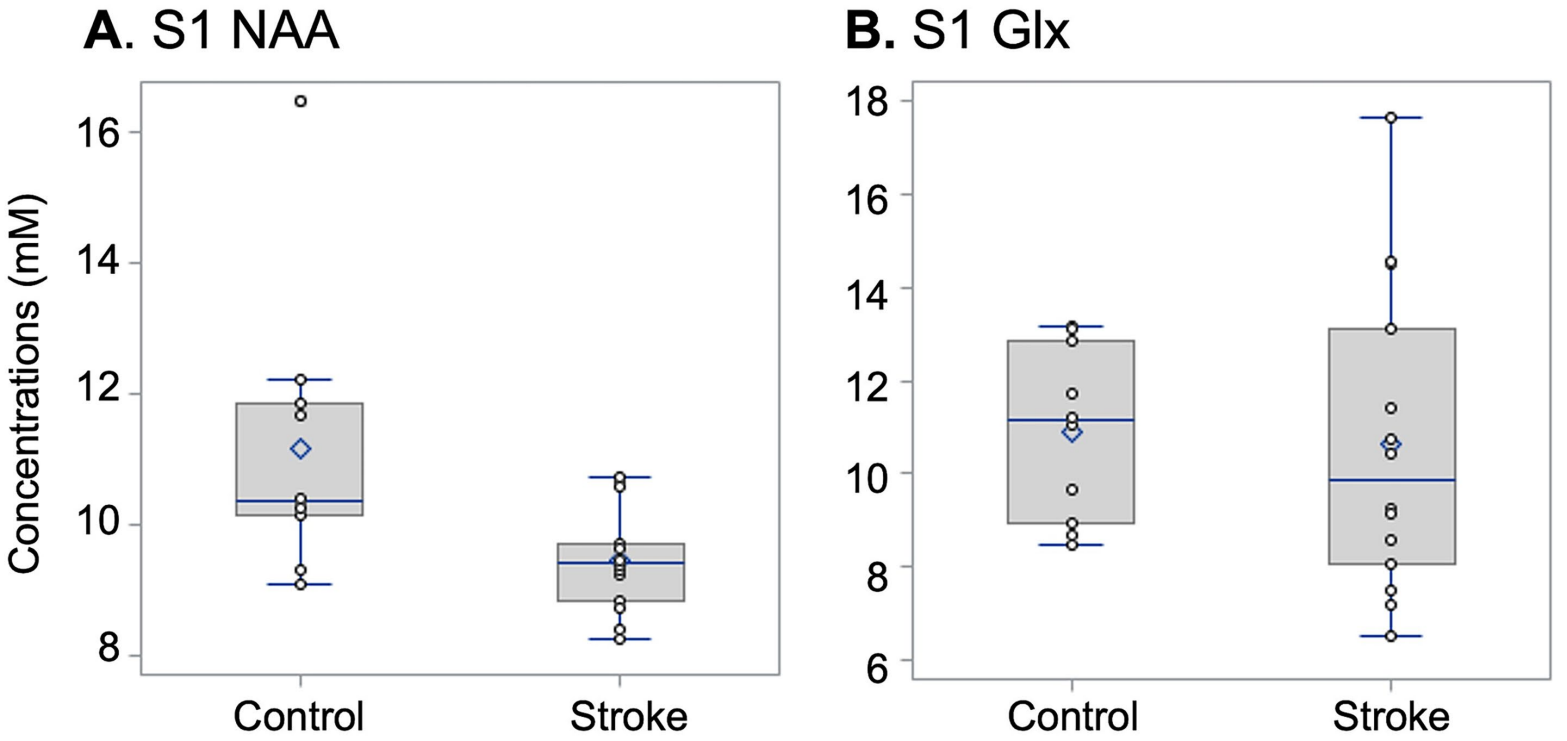
Box and whisker plot (with individual data points, circles) for NAA **(A)** and Glx **(B)** concentrations in left S1 for the control group and in ipsilesional S1 for the stroke group. Between-group differences in NAA reach statistical significance (p=0.01), and the significance remains after removing the outlier from the control group (p=0.02). The boxplot is a visual representation of the interquartile range, showing the first quartile (lower) and third quartile (upper), along with the median (blue line within the box). The whiskers represent the max and min values.

As predicted, patients showed significantly lower NAA levels than controls [*t* = −2.8, estimates (ES) = −1.7, 95% CI: (−2.9, −0.5), *p* = 0.01] (Figure 2A and Table 2). NAA difference remains significant after controlling for age and sex (ES = −1.5, *p* = 0.02), and even after removing the outlier from the control group (ES = −1.1, *p* = 0.009). NAA was significantly lower in the subacute subgroup (ES = −2.1, *p* = 0.02), but not in the chronic subgroup (ES = −1.4, *p* = 0.13) (Table 2; Figure 2).

**Table 2:** S1 NAA and Glx (mean, standard deviation, minimum, maximum) in left S1 in controls and in ipsilesional S1 in patients (the entire group, subacute, and chronic subgroups).

Participants	NAA (mM)	Glx (mM)
Controls	11.2, 2.1, 9.1, 16.5	10.9, 1.8, 8.5, 13.2
Patient group	9.5, 0.8, 8.3, 10.7	10.6, 3.3, 6.5, 17.7
*p*-value	0.01	0.82
Subacute patient subgroup	9.1, 0.4, 8.4, 9.7	9.3, 2.4, 6.5, 13.1
*p*-value	0.02	0.15
Chronic patient subgroup	9.8, 0.9, 8.3, 10.7	11.9, 3.7, 7.5, 17.7
*p*-value	0.13	0.46

*p*-value signifies the difference between controls and patients.

### Stroke participants show no significant difference in Glx levels compared to controls

Contrary to our expectations, the Glx levels were not significantly different in patients vs. controls [*t* = −0.23, ES = −0.26, 95% CI: (−2.5, 2.0), *p =* 0.82] (Figure 2B and Table 2), even after controlling for age and sex (ES = −0.16, *p* = 0.90) or in the subgroup analysis (subacute, ES = −1.54, *p* = 0.15; chronic: ES = 1.02, *p* = 0.46) (Table 2).

### Stroke participants exhibit significant NAA-Glx correlations compared to controls

We found significant correlations between NAA and Glx in the entire patient group (Spearman *r* = 0.60, *p* = 0.02) and subgroups (subacute, *r* = 0.48, *p* = 0.05; chronic, *r* = 0.49, *p* = 0.04), but not in controls (*r* = 0.61, *p* = 0.06).

### S1 NAA levels correlate with hand motor impairment in the chronic stroke subgroup

**Figure 3:**
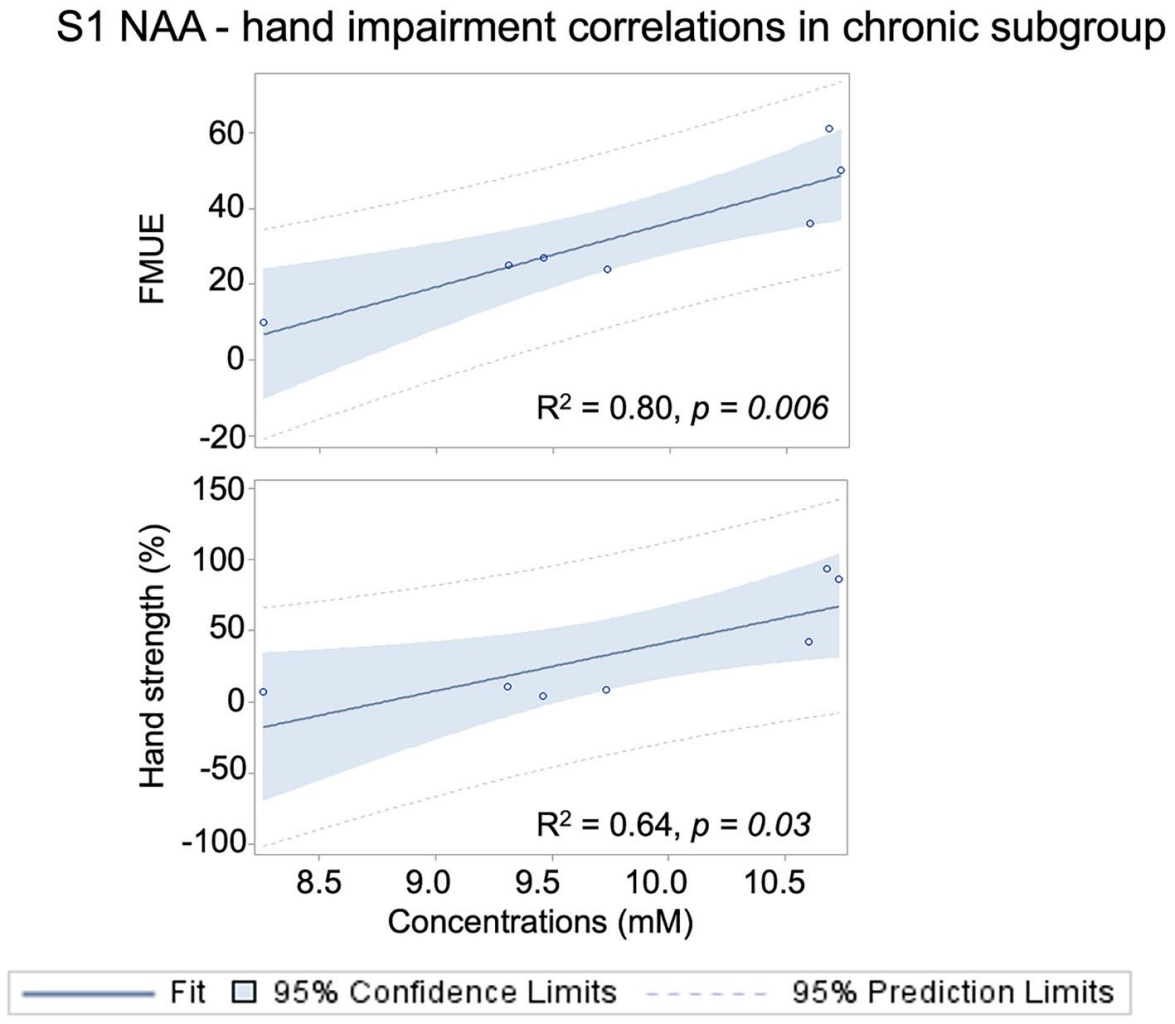
Scatterplots (regression line with 95% confidence limits and 95% prediction limits) show significant positive correlations between ipsilesional S1 NAA concentrations and hand motor impairment (FMUE, top) and strength (Jamar dynamometer, % of the non-paretic hand) in patients with chronic stroke (>6 months after onset).

Significant correlations were found in the chronic subgroup between NAA and FMUE (*R*^2^ = 0.80, *p* = 0.006) and hand strength (*R*^2^ = 0.64, *p* = 0.03), suggesting that higher NAA levels are associated with better hand motor function (Figure 3).

Yet, for the entire group and subacute subgroup, we failed to detect statistically significant correlations between NAA levels and motor impairment clinical scores (Supplementary Table S2). Non-significant correlations were found between Glx and hand impairment in the entire patient group or subgroups (Supplementary Table S2; Supplementary Figure S1).

## Discussion

To our knowledge, this is the first study to provide evidence of altered neuronal integrity in the radiologically normal-appearing S1 in patients with subacute-to-chronic stroke. As predicted, stroke participants exhibited lower levels of NAA, a putative biomarker of neuronal integrity, in the somatosensory paretic hand region, which positively associated with Glx levels in that area. Contrary to our predictions, the NAA levels were not correlated to the clinical level of arm motor impairment in the entire patient group, but in those with chronic stroke. We also failed to find significant between-group/subgroups differences in Glx levels, nor significant correlations between Glx and hand impairment. Below, we discuss these findings in detail, along with their implications and the limitations of our study.

### Lower levels of NAA are found in the S1 hand territory contralateral to the paretic hand

Consistent with our predictions, we found lower NAA levels in the S1 hand territory compared to the analogous territory in matched healthy controls (see Figure 2A). For the entire group of patients, the magnitude of NAA decrease (by 14.5% compared to controls) is similar to those reported in the hand territories of the ipsilesional M1 (lower by 14.2%) (Cirstea et al., 2011) or supplementary motor cortex (13.9%) (Cirstea et al., 2012) in a similar population. Consequently, we consider the differences in NAA levels reported here, relative to those in controls, to be reliable and likely to reflect a similar phenomenon observed in the motor cortices. Lower levels of NAA are thought to indicate neuronal metabolic downregulation and/or neuronal death (see review Moffett et al., 2007). Because the MRS voxels were selected in radiologically intact S1 distant from injury, no thalamic damage was detected, and similar brain tissue composition exists within the selected spectroscopic voxels (Supplementary Table S1), neuronal death appears unlikely. Further, the lack of a significant NAA difference between those with chronic stroke and controls (Table 2) provides additional support for the view that the observed NAA alterations are unlikely to reflect neuronal death. However, due to the small sample size (*n* = 7) in this subgroup, we interpret these findings with caution. Alternatively, lower NAA could be due to decreased cerebral blood flow, likely caused by carotid artery stenosis (Sklinda et al., 2020), which may be present in these patients (and was not evaluated in this study). Such blood flow alteration would be expected to have a more global effect on NAA levels, affecting sensorimotor areas in that hemisphere. Since we did not find altered NAA levels in the ipsilesional dorsal premotor cortex in a very similar population (Cirstea et al., 2012), we speculate that altered blood flow is unlikely to explain our NAA findings. Therefore, lower NAA may instead reflect neuronal metabolic downregulation, likely due to diaschisis (Jia et al., 2026; Pascoa Dos Santos and Verschure, 2021; Seitz et al., 1999). Briefly, diaschisis refers to the suppression of neuronal activity, metabolism, or perfusion in spared brain regions remote from, but connected to, the injury site, resulting from disruption of afferent or efferent pathways. Yet, diaschisis may also contribute to a range of long-term cellular-level metabolic alterations, such as abnormalities in mitochondrial function or decreased metabolic activity; lower NAA supports these changes. This phenomenon could also lead to long-term morphological changes, i.e., shrinkage in body cell size, reduction in the number of synaptic buttons, incomplete endings, and loss of synapses; yet, we did not find significant differences in grey/white matter volume between our patients and controls (Supplementary Table S1). However, we cannot exclude microscopic neuronal changes. Finally, diaschisis could lead to changes in the excitability-inhibition balance; we found non-significant changes in Glx levels in S1 (see detailed discussion below). Still, the positive and significant correlation between NAA and Glx levels indicates a direct relationship between neuronal metabolic status and excitability level, i.e., an altered metabolic status is associated with lower excitability. A similar change in NAA-Glx coupling was observed in the ipsilesional M1 (Cirstea et al., 2011), further supporting our interpretation.

### Glx levels in S1 hand territory contralateral to the paretic hand were not significantly altered

It is important to note that the differences observed between stroke participants and controls were specific to NAA and were not evident for Glx (Figure 2B and Table 2). The lack of significant Glx alterations could be partially explained by differences in cellularity between S1 and M1. Indeed, approximately half of the synapses in M1 are exclusively excitatory, whereas in S1, there are more synapses formed with both excitatory and inhibitory neurons (Bopp et al., 2017). Anatomically, S1 is closely connected with M1, with excitatory projections from S1 to M1 (Papale and Hooks, 2018; Rocco-Donovan et al., 2011) and mostly inhibitory projections from M1 to S1 (Gandolla et al., 2014), enabling complex information integration during sensorimotor control and learning. This S1-M1 infrastructure may be shaped during stroke rehabilitation, and such reshaping may contribute to our findings. Future studies are warranted to determine the extent to which this infrastructure is affected by stroke rehabilitation.

### S1 NAA levels correlate with clinically detected hand/arm impairment in patients with chronic stroke

Contrary to our prediction, we did not find significant correlations between NAA levels and clinical impairment in the entire patient group. Still, those with chronic stroke showed significant, positive correlations between NAA and hand motor impairment (Figure 3). These findings corroborate our prior MRS results in a larger sample of chronic stroke survivors (Cirstea et al., 2011; Cirstea et al., 2012; Craciunas et al., 2013). The failure to find significant correlations between NAA levels and clinical impairment in the subacute subgroup (Supplementary Table S2) could be explained by decreased variability in clinical impairment within this subgroup, i.e., 57% of patients having no hand impairment (FMUE = 66). Yet, it remains unclear whether these neuronal-level changes contribute to the hand motor impairment or are responses to chronic alterations in afferent input to S1 or in M1-S1 connectivity. Obviously, more work is needed to understand the nature of these relationships.

### S1 Glx levels did not correlate with clinically detected hand/arm impairment

We also failed to find significant correlations between Glx levels and arm impairment in the entire patient group or subgroups (Supplementary Table S2). For the entire group, this failure could be explained by the relatively wide time range post-stroke (from 1 month to 12 years), which may mask the role of S1 excitability changes in recovery from the impairment, since initially suppressed ipsilesional S1 excitability increased over time (Carson, 2020; Williamson et al., 2024). For subgroups, a potential reason for this failure could be the clinical tests’ low sensitivity to capture changes in excitability, possibly related to the sensory-motor integration. More work with larger samples and more sensitive testing is warranted.

### The current study has several limitations

The first limitation is the sample size; although comparable to prior MRS studies in this population, it limits statistical power and may have prevented the detection of subtle effects. Second, the variance of time since stroke is relatively large. Yet, we analyzed the entire patient group and two subgroups based on chronicity, and we also corrected the correlation analyses for the time after stroke onset. Third, although no deficits in tactile discrimination (light touch assessed with Semmes-Weinstein monofilaments) were found, a more systematic evaluation of the S1 function, i.e., proprioception, pain, vibration, would help us better understand the relationship between S1 metabolic changes and sensorimotor function.

## Conclusion

In summary, we found altered neuronal integrity in the spared S1 hand territory months to years after a unilateral subcortical stroke. The robust coupling between neuronal integrity and cortical excitability across all patients and between neuronal integrity and hand motor impairment in those with chronic stroke provides a means to assess the fine-grained underpinnings of possible diaschisis in S1. Understanding how stroke impacts sensory information processing and/or flow in different stages of stroke will help design effective therapeutics for tackling a major contributor to disability after stroke, the hand motor impairment.

## Data Availability

The raw data supporting the conclusions of this article will be made available by the authors, without undue reservation.
