# Supplementary material for: Somatosensory cortex neuronal integrity is altered after stroke
Source: Front Hum Neurosci. 2026 May 20;20:1810922. doi: 10.3389/fnhum.2026.1810922 (PMC13230184; doi:10.3389/fnhum.2026.1810922)
Supplement: Supplementary file 1 [file Table_1.DOCX]

**Table 1S.** Signal-to-noise (S/N) and spectroscopic grey and white matter tissue composition (%) in individual participants.

| **Participants** | **S/N** | **Voxel GM + WM** |
| --- | --- | --- |
| **Controls** | | |
| 1 | 11 | 96.2 |
| 2 | 14 | 81.2 |
| 3 | 11 | 83.4 |
| 4 | 15 | 75.6 |
| 5 | 14 | 94.7 |
| 6 | 11 | 87.3 |
| 7 | 11 | 89.3 |
| 8 | 13 | 90.2 |
| 9 | 14 | 84.5 |
| 10 | 13 | 80.9 |
| **Stroke** | | |
| 1 | 11 | 83.4 |
| 2 | 11 | 93.3 |
| 3 | 12 | 89.1 |
| 4 | 11 | 92.8 |
| 5 | 11 | 91.2 |
| 6 | 10 | 84.3 |
| 7 | 11 | 93.5 |
| 8 | 12 | 91.3 |
| 9 | 11 | 85.9 |
| 10 | 11 | 84.6 |
| 11 | 15 | 90.3 |
| 12 | 14 | 89.5 |
| 13 | 12 | 93.2 |
| 14 | 10 | 98.9 |

**Table 2S.** Correlation analysis (R^2^, *p-value*) between ipsilesional S1 NAA and Glx and hand impairment (FMUE and hand strength) in the entire patient group and subacute subgroup.

| **Correlations** | **Patient group** | | **Subacute group** |
| --- | --- | --- | --- |
|  |  | Controlling for time post-stroke |  |
| NAA-FMUE | 0.0001, *0.97* | 0.24, 0.*87* | 0.20, *0.31* |
| NAA-Hand strength | 0.001, *0.89* | 0.25, 0.*78* | 0.28, *0.22* |
| Glx-FMUE | 0.02, *0.61* | 0.26, 0.*61* | 0.07, *0.57* |
| Glx-Hand strength | 0.000, *0.99* | 0.25, 0.*96* | 0.02, *0.78* |
