# Supplementary figures and images for: Somatosensory cortex neuronal integrity is altered after stroke
Source: Front Hum Neurosci. 2026 May 20;20:1810922. doi: 10.3389/fnhum.2026.1810922 (PMC13230184; doi:10.3389/fnhum.2026.1810922)

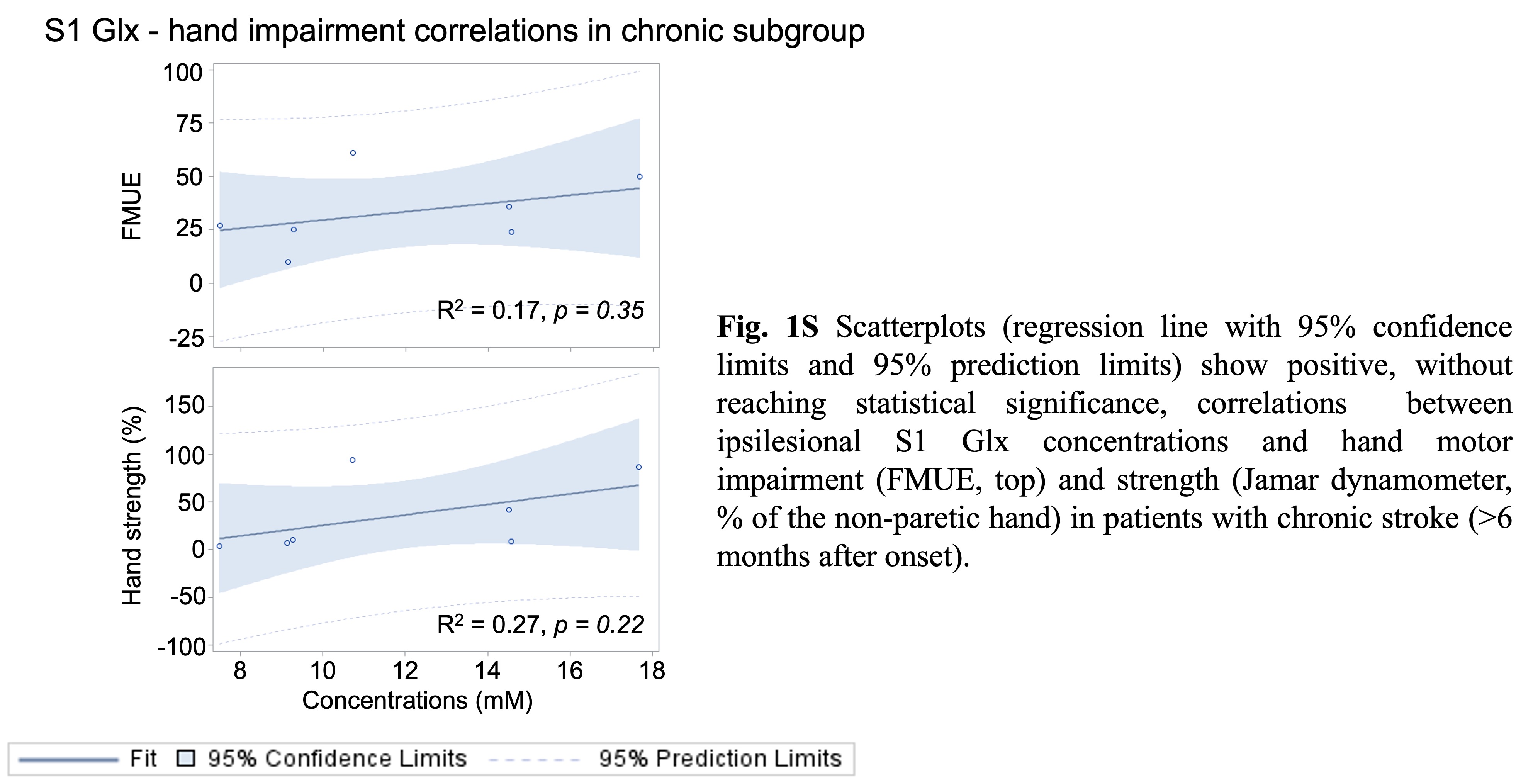

Supplement: Supplementary file 2 [file Image_1.JPEG]
